# Gut microbiota: in sickness and in health

**DOI:** 10.1111/1751-7915.12106

**Published:** 2014-01-05

**Authors:** M José Huertas, Carmen Michán

**Affiliations:** 1 Instituto de Bioquímica Vegetal y Fotosíntesis, Centro de Investigaciones Isla de la Cartuja, Universidad de Sevilla-CSICAv. Américo Vespucio 49, 41092, Seville, Spain; 2 Campus de Rabanales, Department of Biochemistry and Molecular Biology, Universidad de CórdobaEdificio Severo Ochoa C-6, 2a Planta, 14071, Córdoba, Spain

At birth, the human colon is rapidly colonized by a vast amount of gut microbes that affect host physiology and metabolism because of their ability to ferment nutrients and secrete bioactive compounds (de Vos, 2013). Lately, different reports are exploring their potential use for the treatment of a wide variety of diseases, although the clinical use of the gut microbiota is not all that innovative, since detailed faecal therapies for the treatment of abdominal pains were documented by Ming dynasty Chinese doctors, over 1000 years ago (Zhang *et al*., 2012). Researchers are now working on how faecal micro-organisms contribute to diseases, particularly in the context of obesity and other related metabolic disorders or ageing.

Several publications analyse the differences in composition and/or activity of the gut microbiota of slim vs. fat individuals, proposing that only few bacterial genera are associated with obesity, swelling and associated metabolic disorders, both in humans and rodents. But the main difficulty in determining these relationships is the fluctuation of microbial community configuration among unrelated individuals. We highlight an article published in *Science* (Ridaura *et al*., 2013) because of its smart approach. The authors describe the transplantation of faecal microbiota from several pairs of human twins, an obese and a slim one, into germ-free mice. The rodents that received the obese twin's faecal microbiota noticeably increased their adipose mass compared with the receptors of the slim twin's microbiota. Furthermore, cohousing of mice harbouring ‘obese’ microbiota along with mice harbouring a ‘slim’ one, prevented high body mass and obesity in the first group, which the authors attributed to a directional transfer of microbes. Also, this invasion of beneficial bacteria seems to depend on food intake because obesity was not prevented when obese mice harbouring a ‘slim’ microbiota ate a high-fat/low-fibre diet (Ridaura *et al*., 2013).

The importance of diet on the establishment of the gut flora meets the results of a long-term study on intestinal microbiota published in *Environmental Microbiology* showing that, although adults gut microbiota profiles are unique and mostly stable, diet, together with the use of antibiotics, intestinal transit and lifestyle clearly alter their proportions (Rajilić-Stojanović *et al*., 2013). These authors designed a comprehensible and highly reproducible phylogenetic microarray, the human intestinal tract chip, to analyse faecal samples of young adults gathered for several years and identifying subject-specific microbiota profiles preserved over time. They also showed how changes in environmental conditions are associated with changes in the abundance of certain species/genera of this microbiota. The challenge now is to link specific metabolic disorders to their corresponding gut microbiota alterations.

This question has also been addressed in a *Nature* article that reports the correlation between diversity of gut microbial genes, metabolic markers (e.g., body weight, fat mass, glucose and lipid metabolism, inflammation) and susceptibility to lose weight upon dietary restrictions (Cotillard *et al*., 2013). Using a quantitative metagenomics approach, Cotillard *et al*. proposed a bimodal distribution of microbial genes: low gene count (LGC) and high gene count (HGC). Individuals with a LGC showed increased weight, adiposity, insulin resistance and inflammatory diseases over time compared with HGC individuals. Additionally, and in agreement with the work by (Rajilić-Stojanović *et al*., 2013), individuals with a LGC also exhibited specific microbial genera known to be linked to inflammatory diseases or altered gut barrier function. The authors concluded that individuals with LGC exhibited metabolic disorders and suggested that microbial gene wealth may be enhanced by dietary intervention. In the near future, these parameters may be used as biomarkers for the prognosis of obesity-related diseases.

Another current issue is the relationship between gut microbiota and human ageing that Harald Brüssow has recently reviewed in *Microbial Biotechnology*, in view of the classic book ‘*The Prolongation of Life*’ by Metchnikoff (1907), and the ELDERMET project that pursues health improvement of elderly people via modification of their gut flora (Brüssow, 2013a). All the reviewed studies show constraints that prevent from drawing a clear conclusion: small samples, high inter-individual variability, changing nutritional habits among populations and/or along life or detecting the presence of bacteria but not their viability. Nevertheless, the decay of gut microbiota in elderly people seems to be more related with poor diet habits and lack of exercise, than with ageing itself. All of that work will facilitate targeted modifications on the microbiome structure – maybe via specific food intake – that will help to improve gut communities in the elderly as suggested by (http://eldermet.ucc.ie/). Complementary to this report and also in *Microbial Biotechnology*, Prof. Brüssow presents an inquisitive review of the different health concepts (Brüssow, 2013b), and discusses whether it can be measured with the appropriate tools. The author also examines the concept of ageing – not only in humans – and how our current knowledge may be biased by short-lived animal studies. Although so far our life expectancy keeps growing, biological systems are not perfect, and so they will probably come to a point where accumulated errors will no longer allow life, particularly on complex organisms. If the focus is not set on increasing our life expectancy, but rather on improving the quality of our ageing, it would be a better choice, and the study of the gut microbiota will undoubtedly contribute to that improvement.

Faecal communities are crucial for health not only in adults or seniors, but also in children, because besides their common functions, they play an important role in the development of their immune system (Jost *et al*., 2012). Neonates' initial colonization depends both on transference from their mothers and on their environment, either during birth or through close contact. Benefits from breastfeeding include the acquisition of probiotic bacteria, but also of other anaerobic microbes that have not been extensively investigated yet. Jost and co-workers have published a very revealing paper in *Environmental Microbiology* (Jost *et al*., 2013), establishing the correlation between micro-organisms found in maternal faeces, breast milk and the gut of their corresponding neonates. The novelty of this report is that the authors not only address the presence of the micro-organisms but also their viability, dealing with one of the deficiencies commonly found in gut communities studies. Their results clearly show divergences between both approaches. Pyrosequencing assigns similar numbers to both mothers' milk and faeces bacteria, but when determining ribosomal ribonucleic acid or colony-forming unit counts, milk samples contain more than a million-fold less microbes than faecal ones. This report certainly supports the transference of living gut microbes from mothers to infants through breastfeeding, although the route between the mother's intestines and the breasts still remains unknown.

Where does all this research lead to? Maybe the clue is to be found in a recently published *Microbial Biotechnology* review by Willem de Vos where he highlights the need to understand how intestinal microbial communities work to develop effective biotechnological tools for targeted modification of gut flora (de Vos, 2013). He establishes the concept of minimal microbiome that can be defined as the smallest set of microbes and/or microbial functions necessary to develop a stable community. Systemic analysis of the interaction between intestinal microbes and the host are under way and will allow the prediction of the minimal synthetic microbiomes for the treatment of different diseases. Next-generation therapies against obesity, ageing and metabolic disorders could be based on the targeted implementation of these microbial communities.
